# Community Composition of Epibiont Hydroids of the Naturalized Alien Macroalga *Acanthophora spicifera* in Pichilingue, Mexico

**DOI:** 10.3390/biology14010044

**Published:** 2025-01-08

**Authors:** Jessica Licona Angeles, Francisco Rubén Castañeda Rivero, María A. Mendoza-Becerril

**Affiliations:** 1 Facultad de Estudios Superiores Iztacala, Universidad Nacional Autónoma de México (UNAM), Av. de los Barrios 1, Tlalnepantla de Baz 54090, Estado de México, Mexico; jessliconamars@gmail.com; 2 Departamento de Áreas Naturales Protegidas Zona Sur, Instituto de Biodiversidad y Áreas Naturales Protegidas del Estado de Quintana Roo (IBANQROO), Hidroeléctrica de Malpaso 398, Campestre, Chetumal 77030, Quintana Roo, Mexico; fruben.castaneda@gmail.com; 3 Departamento de Sistemática y Ecología Acuática, El Colegio de la Frontera Sur (ECOSUR), Av. Centenario km 5.5, Pacto Obrero, Chetumal 77014, Quintana Roo, Mexico

**Keywords:** epibiosis, hydrozoa, La Paz Bay, Mexican Pacific, rhodophyta

## Abstract

This study provides a detailed analysis of the community of hydrozoan epibionts of *Acanthophora spicifera* in La Paz Bay, Baja California Sur. The investigation was carried out in two season periods, corresponding to the warm and cold seasons on the pier of the Autonomous University of Baja California Sur (UABCS) Pichilingue research station. The percentage of epibiont coverage was calculated and analyzed. The average length of the thalli was 7.8 ± 4.6 cm. Eleven taxa of hydrozoans were recorded; the hydrozoan *Obelia* cf. *dichotoma* had the highest coverage percentage, and in summer, there was a higher taxa richness. It is recommended to monitor this macroalga and its epibionts for its wide distribution in the bay, although in UABCS, Pichilingue Pier has not seen epibionts that threaten biological diversity.

## Simple Summary

The red macroalgae *Acanthophora spicifera* (spiny seaweed) is a naturalized alien species at La Paz Bay and appears to tolerate areas with human activity. However, before this study, we were unaware of the type and quantity of hydrozoan epibiont growing on this alga. Therefore, this study aims to examine the community structure of hydrozoan epibionts of *A. spicifera* in warm and cold seasons. We recorded eleven hydrozoans, of which there are more in summer.

## 1. Introduction

Marine macroalgae are among the most conspicuous and familiar organisms of the marine coastal shelf environment; they manifest in various forms, from small, rock-like turfs encrusting intertidal rocks or tide pools to immense subtidal kelps that wash up on exposed beaches [1]. Therefore, macroalgae significantly influence marine fauna’s distribution, diversity, and abundance [2] and provide a substrate by creating microhabitats where epibionts can attach, grow, and reproduce [3]. However, few studies analyze their basibiont role and impact on coastal marine ecosystems.

Of all the studies on hydrozoan epibionts of non-native or invasive macroalgae that have been developed worldwide (e.g., [4,5,6,7,8,9,10,11]), and more specifically, in Mexico (e.g., [12,13,14,15]), seasonality has not been addressed to date in detail. The diversity and abundance of invertebrate epibionts of macroalgae, in general, vary seasonally. For example, the abundance of amphipods and ostracods is strongly influenced by the changing seasons [16], and the compositions, occurrence, and abundance of hydroids are also influenced by seasonal cycles [17,18].

Of non-native or introduced macroalgae species, *Acanthophora spicifera* (M. Vahl) Børgesen has been considered an alien species in the Pacific Ocean since its discovery in 1952 on Hawaii’s coasts [19] and as a naturalized alien species without evidence of a negative impact in La Paz Bay, Mexican Pacific [13]. However, it has been observed that there is a preference for sites with anthropogenic activity, where the highest presence of epibionts (bryozoans and hydrozoans) has also been recorded [13]. This seaweed colonizes mainly hard substrata from the intertidal to 0–10 m depth, such as rocks, mussels, sponges, coral rubble, artificial substrates, and, to a lesser extent, sandy substrates [13,20].

There are no reports on the threat this naturalized alien species poses to native wildlife, ecosystem functioning, the economy, or human health in La Paz Bay. Nor has it been possible to link this disperser to any specific invasion of other invertebrates, such as hydrozoans, due to the lack of a baseline of hydrozoan diversity in the bay before this introduction, as well as the lack of monitoring of *A. spicifera* and their epibionts since their first record in the region. Therefore, it is important to identify and inventory the epibiont species growing on introduced macroalgae to monitor and manage the impact on local biodiversity [14]. In this context, the main objective of this study is to examine the community structure of the hydrozoan epibionts of *A. spicifera* in warm and cold seasons at the disturbed locality of La Paz Bay, the pier of the Autonomous University of Baja California Sur (UABCS) Pichilingue research station, as well as, to suggest a sampling design of monitoring for macroalgae and its hydrozoan epibionts for La Paz Bay.

## 2. Materials and Methods

### 2.1. Sample Collection

**Figure 1:**
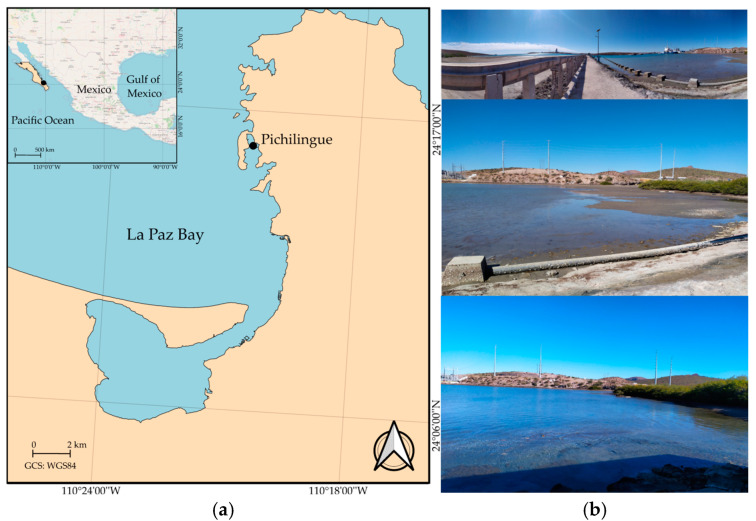
Study area: (**a**) UABCS Pichilingue Pier, La Paz Bay, Baja California Sur, Mexico; (**b**) front and side views of the UABCS Pichilingue Pier beach.

Pichilingue’s port is situated in La Paz Bay in the southwestern region of the Gulf of California, Mexico. In La Paz Bay, two seasons are clearly distinguished during the year: a cold season with an average temperature range of 20.5–26.0 °C from December to May (cold waters) and a warm season of 26.0–31.0 °C from June to November (warm waters) [21,22,23]. The Pichilingue port creates a false bay that can accommodate diverse maritime traffic, including cargo ships, fishing boats, and large ferryboats [24]. To the southeast of this bay is the pier of the Autonomous University of Baja California Sur (UABCS) Pichilingue research station (24°16′12.0″ N, 110°19′30.0″ W; Figure 1). This research station’s beach has a predominantly sandy substrate, with some muddy areas resulting from mangroves in the surrounding area. Within the interior of the beach, macroalgal aggregations adhere to rocks, the sandy bottom, and anthropogenic structures such as the pier’s buoys. The depth of the water over the sand oscillates from 40 cm near the pier on the rocky shore to 170 cm in the central and western regions of the beach. The western region of the beach is lined with buoys covered with *A. spicifera* macroalgae and other encrusting organisms, including ascidians, sponges, and bryozoans.

The entire study area, extending from the beach to the buoy zone, was surveyed by diving and snorkeling at a depth range of 0–7 m. A total of 60 thalli of the rhodophyte macroalga *A. spicifera* were only located on float buoys and rope floats and collected manually by scraping in July 2021 (warm season) and February 2022 (cold season) (Table S1). All thalli collected were fixed in 96% ethanol for hydrozoan epibionts’ identification. The temperature and salinity were measured in situ.

### 2.2. Sample Processing

The thalli were identified according to De Jong et al. [25] and examined in the laboratory. The total length of each stem was measured, and hydrozoan presence or absence was recorded using the microscopes ZeissStemi 2000-C and Zeiss Axio Scope A1 (Göttingen, Germany). Epibionts (specimens that exhibited suitable morphological conditions, for example, with a hydrorhiza, stem, and hydranth) were identified with the support of taxonomic descriptions and compilations available in the literature (e.g., Calder [26,27,28], Mendoza-Becerril et al. [14,29]). The nomenclature used was based on a study by Maronna et al. [30] for the Leptothecata hydroids and Ahyong et al. [31] for other hydroids. After the analysis, the specimens (algae and hydrozoans) were kept in the macroalgae laboratory at Centro de Investigaciones Biológicas del Noroeste, S.C.

On each *A. spicifera* thallus, the hydrozoan species cover (as a measure of abundance) was determined following the procedures described by Cunha and Jacobucci [17] and Mendoza-Becerril et al. [18]. The hydroid coverage on each thallus was measured, considering the hydrorhiza of the hydroids in contact with the thallus. For this, the thallus was extended in its entirety between acrylic plates, and the number of squares occupied by both macroalgae and hydrorhiza on both sides of the plates was counted, taking into account the three-dimensionality of the thalli.

**Figure 2:**
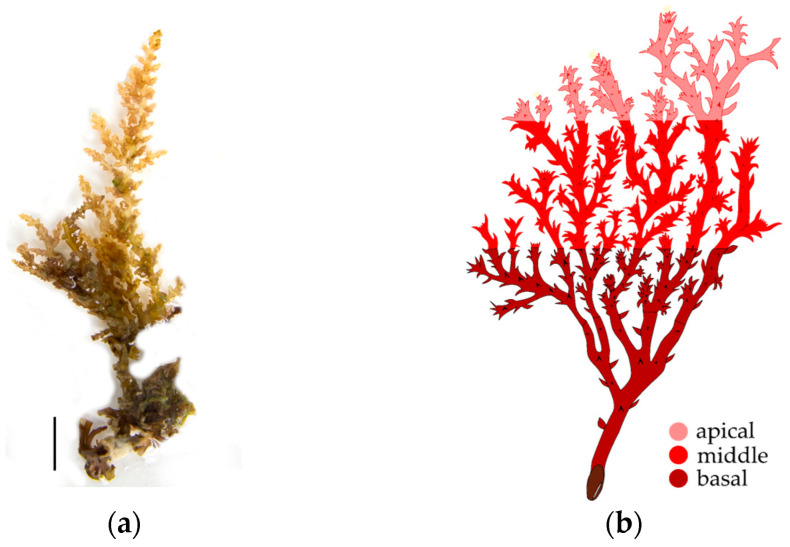
*Acanthophora spicifera* of UABCS Pichilingue Pier, La Paz Bay, Baja California Sur, Mexico: (**a**) Fiel sample, scale equals 1.0 cm, photo: I. Domínguez Guerrero; and (**b**) scheme with sections for recording hydrozoans.

Additionally, we observed the presence or absence of hydrozoans on each thallus section, to which each thallus was segmented into three equal parts: the basal section consisted of the first third closest to the disc and part of the stem, the middle section included the central part of the alga, and the last third of the thallus from the middle part to the tips of the alga was cataloged as the apical section (Figure 2).

### 2.3. Data Analysis

To evaluate the sampling effort, taxa accumulation curves were built with 1000 randomizations without replacement, considering all thalli, and evaluated with the following non-parametric estimators: Chao1, Chao2, and Bootstrap using the PRIMER program version 7 [32]. To compare the taxa richness, we interpolated and extrapolated curves of estimated taxa richness per season and thallus sections in relation to the cover with 95% confidence intervals obtained with 1000 randomizations, using the “iNEXT” function of the “iNEXT” package [33]. Richness was standardized based on the smallest sample. In addition, sample completeness (as measured by the sample coverages) was obtained for each climatic season and thallus section (Sc = the proportion of the number of individuals or total coverages in the community belonging to the species represented in the sample) [33].

Our analyses included only thalli with the presence of hydrozoans identified to species. An Olmstead–Tukey diagram [34] was used to classify dominant, occasional, frequent, and rare species based on the percentage coverages of each hydrozoan species [35]. In addition, the biological value index (BVI) was calculated for the season and thallus sections [36]. Data were analyzed using the R software libraries version 2024.04.1+748 [37], and for visualization, the GGPLOT2 package version 3.5.1 was used [38]. A Venn diagram was constructed to identify exclusive hydrozoan species for the season and thallus sections, employing the EULERR package [39].

The non-parametric Kruskal–Wallis test was performed (normality and homoscedasticity of the a priori cover data were not present) using the R STATS package [37] and the PGIRMESS package [40] to compare the changes in the percent cover of the hydroids by season and thallus section. For this analysis, only thalli with hydroids in only one section were considered; those with epibionts in more than one section were not considered. A linear regression model was employed to ascertain whether there is a correlation between the size of the collected thalli and the percentage of coverage of the identified hydroids, using the R STATS package [37], and for visualization, the GGPLOT2 package was used [38].

To identify similarities in the community structure of the hydrozoans between seasons and sections (hydrozoan coverage on thalli), an nMDS was performed using the Bray–Curtis method [41] with the prior transformation of the fourth root (∜) to reduce the influence of dominant taxa on the percentage coverages of each hydrozoan species [42], using the PRIMER program version 7 [32].

## 3. Results

The average length of the thalli was 7.8 ± 4.6 cm, with 87% ≤ 10 cm and 85% presenting hydrozoan epibionts (*N* = 51); the average length in the warm season was 6.4 ± 1.9 cm, while the cold season was 8.3 ± 2.3 cm. Eleven taxa of hydrozoans were recorded (Table 1), most of which corresponded to one taxa per thallus, except for five thalli that presented two taxa. The hydrozoan with the highest coverage percentages in each season was *Obelia* cf. *dichotoma*. The total coverage was 73.3 in the warm and 15.9 in the cold seasons.

**Figure 3:**
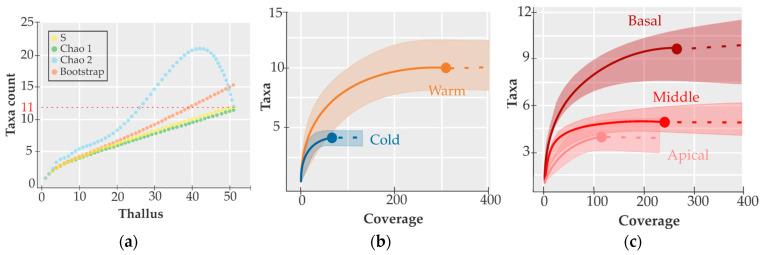
Sampling effort and taxa richness: (**a**) Sampling effort, the taxa-accumulation curve for 51 thalli, Chao 1, Chao 2, and Bootstrap; (**b**,**c**) taxa richness of hydrozoans, sample-size-based interpolation and extrapolation curves of taxa richness with 95% confidence intervals (shaded areas); (**b**) for each season; and (**c**) for each thallus section.

The hydrozoan sampling effort allowed the recording of about 95% of the estimated richness of Chao1 (*S* = 11.5), 100% of the estimated richness for Chao2 (*S* = 12), and 80% of the estimated richness with Bootstrap (*S* = 15). The sample completeness obtained for the two seasons and by the thallus sections collected was high, representing a Sc > 87%, so the recorded species inventory can be considered complete in this habitat (Figure 3a). The warm season registered the highest number of hydrozoan taxa, with ten, compared to the cold season, with four taxa. However, their expected taxa richness (*^0^D* = 6) over the cold season (*^0^D* = 4) was not statistically significant, showing no difference in taxa composition (Figure 3b). The basal section of the thallus presented the highest number of taxa (ten taxa), followed by the middle and apical sections with five and four taxa, respectively (Figure 3c). The expected richness of the basal section (*^0^D* = 7.3) was statistically different from that of the middle (*^0^D* = 4.6) and apical sections (*^0^D* = 4), showing to be different with respect to the low expected richness because their confidence intervals did not overlap (Figure 3c). The hydrozoan epibionts were mainly recorded in the basal section in the warm season and equally in the basal and middle sections in the cold season.

**Figure 4:**
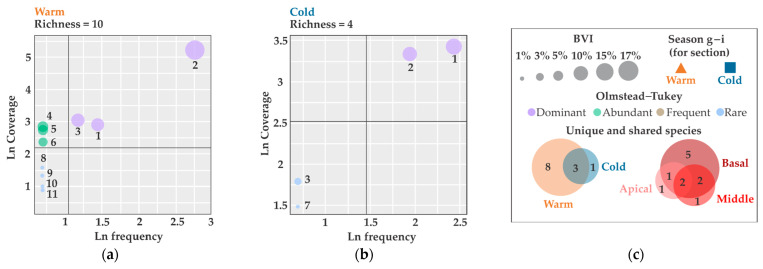

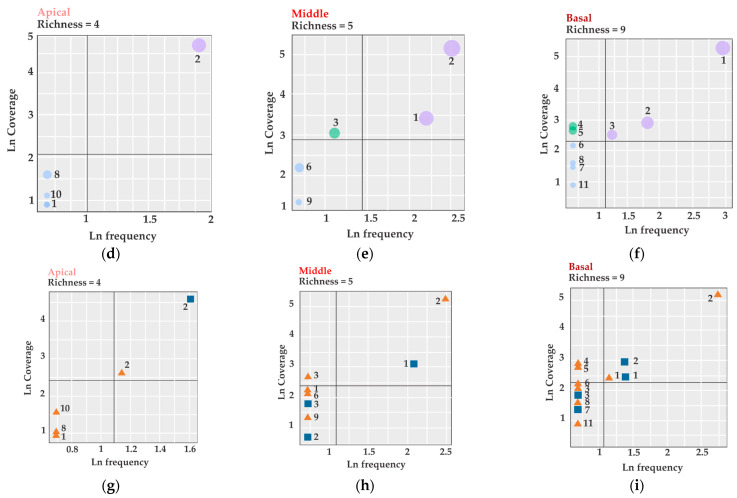
Olmstead–Tukey test: (**a**,**b**) For each season; (**c**) symbols on the chart and Venn diagram illustrating shared hydrozoan taxa between season and section; (**d**–**f**) for each thallus section; (**g**–**i**) for each season and thallus section. ID taxa: 1. *Clytia linearis*, 2. *Obelia* cf. *dichotoma*, 3. *Bougainvillia muscus*, 4. Capitata (Indet.), 5. *Turritopsis* sp., 6. *Ventromma halecioides*, 7. *Corydendrium* sp., 8. *Obelia oxydentata*, 9. *Lafoeidae* (Indet.), 10. *Clytia* cf. *gracilis*, and 11. *Bimeria vestita*.

The Olmstead–Tukey test demonstrated that only three taxa were identified as dominant (27%), while six taxa were classified as rare (55%). During the warm season, three dominant, three abundant, four rare, and eight exclusive taxa were observed, whereas the cold season exhibited two dominant, two rare, and a single exclusive taxon (Figure 4a–c). With regard to the thallus sections, the apical section exhibited the lowest taxa number, comprising one dominant, three rare, and one exclusive taxa; this was followed by the middle section, which showed two dominant, one abundant, two rare, and one exclusive species. Finally, the basal section presented three dominant, two abundant, five rare, and five exclusive taxa (Figure 4d–f). In the case of seasons and thallus sections, *Clytia linearis* and *O.* cf. *dichotoma* were identified as having a particularly high contribution of cover in the samples and were therefore classified as dominant, as well as Capitata (Indet.) and *Turritopsis* sp. as abundant, and *Corydendrium* sp., *Obelia oxydentata*, Lafoeidae (Indet.), *Clytia* cf. *gracilis*, and *Bimeria vestita* as rare species due to their low cover and frequency of occurrence (Figure 4g–i).

**Figure 5:**
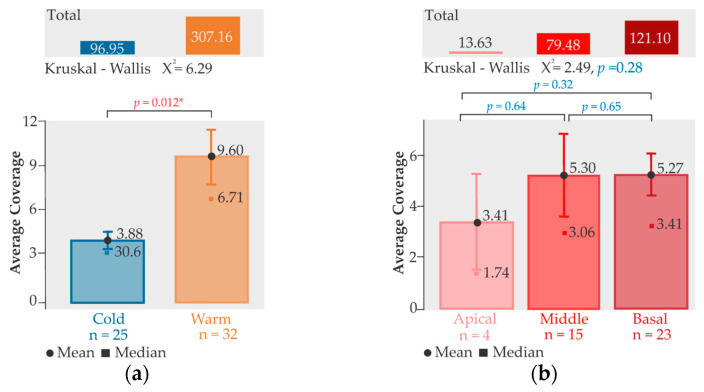
Non-parametric Kruskal–Wallis test: (**a**) For each season; and (**b**) for each thallus section. * *p* < 0.05.

**Figure 6:**
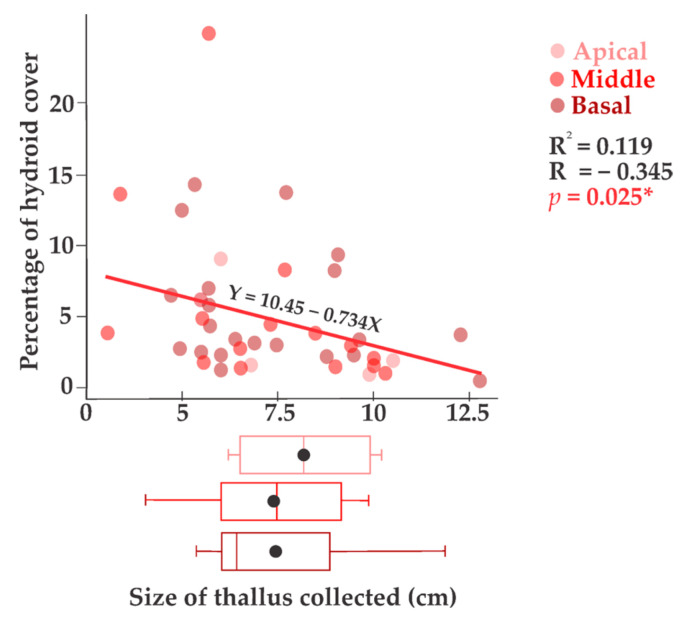
Percentage of hydroid cover (±standard error) on thalli. * *p* < 0.05.

The non-parametric Kruskal–Wallis test showed that there were significant differences between the medians of hydrozoan cover in each season, with a high value for the total and average cover in the warm season (Figure 5a). In contrast, there were no significant differences between the medians of hydrozoan cover based on the three thallus sections (Figure 5b). Considering the total number of thalli collected, a significant (*p* < 0.025) linear and negative relationship was found between the percentage of hydroid cover and the size of the thallus collected (cm), with a slightly lower degree of relationship (*R* = 0.30) (Figure 6).

**Figure 7:**
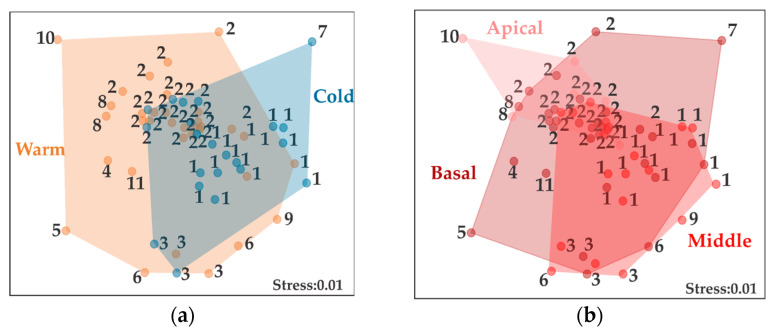
Nonmetric multidimensional scaling (nMDS) showing the similitudes in the community structure of hydrozoans on *Acanthophora spicifera*: (**a**) Hydrozoan cover per season; (**b**) Hydrozoan coverage per section. ID taxa: 1. *Clytia linearis*, 2. *Obelia* cf. *dichotoma*, 3. *Bougainvillia muscus*, 4. Capitata (Indet.), 5. *Turritopsis* sp., 6. *Ventromma halecioides*, 7. *Corydendrium* sp., 8. *Obelia oxydentata*, 9. *Lafoeidae* (Indet.), 10. *Clytia* cf. *gracilis*, and 11. *Bimeria vestita*.

The nMDS ordination analysis based on the hydrozoan community structure showed that most of the cold season thalli were very similar to the warm season thalli, with an overlap observed in those where the taxa *C. linearis*, *O*. cf. *dichotoma,* and *B. muscus* are present (Figure 7a). The thallus sections showed almost complete similarity in community structure (Figure 7b). The apical section differs by only one taxon (*C*. cf. *gracilis*), and the basal section shows taxa that do not overlap with the other two sections (Capitata, *Corydendrium* sp., *Turritopsis* sp., and *B. vestita*) (Figure 7b).

## 4. Discussion

Eleven taxa were identified, of which seven are new records (Capitata Indet., *Corydendrium* sp., *B. vestita*, *B. muscus*, Lafoeidae Indet., *O. oxydentata*, and *Turritopsis* sp.) on *A. spicifera*. Before this study, the maximum number of taxa recorded on this macroalga was 14 worldwide, with 4 taxa shared (*C.* cf. *gracilis*, *C. linearis*, *O.* cf. *dichotoma,* and *V. halecioides*) [9,10]. Therefore, the epibiont hydroids found in this rhodophyte amount to 21 species worldwide. Of the non-native macroalgae species in the world, this has the most epibiont hydroids taxa, followed by *Sargassum muticum,* with 14 taxa recorded [5,43,44].

All hydrozoan taxa epibionts had already been recorded in La Paz Bay and the Mexican Pacific (cf. Estrada-González et al. [45]). The taxa *C. linearis* and *O.* cf. *dichotoma* are common species in the community of hydroids in La Paz Bay [13,14,29,46,47] and are widely distributed in the Mexican Pacific Ocean [45]. These taxa were dominants and presented gonophores, which are characteristics of pioneer and early successional hydrozoans, often encountered in disturbed sites and fouling communities [48]. Even when *O.* cf. *dichotoma* is mentioned in the literature as an invasive species [49,50], no negative damage to the diversity of the localities where it has been recorded has been documented. However, this species exhibits recognized morphological variability and cryptic lineages [51]. Therefore, it is important to carry out a genetic analysis and then confirm its identity before proposing to change its status to a naturalized alien. 

A richness variation between seasons was observed; however, this variation was not statistically significant (overlapped shaded areas, see Figure 3b), but it was significant (not overlapping shaded areas between basal sections with respect to the apical and middle sections, see Figure 3c) in the thallus section and hydrozoan average cover by season (*p* = 0.012). These variations have also been observed in other macroalgae species and different localities (e.g., [15,17,18,52,53,54]). In Brazil, the highest richness was in the cold season, while the highest cover was in the warm season [17], in contrast to what was observed in the Mexican Caribbean, where the highest richness (six species) was in the warm season and covered (52.8%) in the cold season [18]. The observed variability might be caused by the variations in the life cycle and structural complexity of the macroalgae, which influence the hydrozoan’s ability to colonize different sections of the thallus and provide more or less available space for attachment during each season [17,53,54,55]. Variations in environmental conditions and competition also play a role in the establishment, survival, and growth of hydrozoans [56], which is more likely to occur since *A. spicifera* is present all year round in La Paz Bay, and its larger size and biomass [57] did not coincide with a higher cover reported in this study.

The basal section tends to have the highest number of species, decreasing in abundance and number towards the distal sections [15,58]. This basal section can contribute to higher survival and reproductive success of the hydroids, factors that influence the diversity of these organisms [59], compared to the middle and apical sections, where the thalli undergo more active growth and are more exposed to environmental changes over time [13,52,53], for example, local wind, tide currents, and waves to which the Pichilingue zone is exposed [60,61]. This exposure may also explain why only thecate hydrozoan taxa had been recorded in the apical zone, which usually survive such adverse environmental conditions [62,63]. In other red macroalgae, an increase in water flow results in a greater loss or breakage of the epibiont [64]. In contrast, dominant hydroid species such as *O.* cf. *dichotoma* may influence the spatial distribution of other hydrozoans growing on the same macroalga through interspecific competition [65].

Unlike artificial substrates, living algae change in size and shape as they grow and age, as well as the structure of the surrounding community of other organisms and the abiotic conditions they are exposed to [64]. At UABCS’ Pichilingue Pier, the macroalgae *A. spicifera* growing on buoys turned out to be smaller than those observed on sandy bottoms (e.g., 12.1 cm at San Juan de la Costa [13]) and hard substrates (e.g., 16.3 cm at Punta Roca Caimancito [57]). This difference in size may be associated with the fact that the buoys are a more variable habitat, where the macroalgae compete for space with other encrusting organisms (e.g., ascidians and bryozoans); moreover, they are more exposed to the effect of wave action and cleaning of buoys [66].

Despite the difference in the length of the thallus, our study reveals that even in small thalli, the hydrozoan epibiont richness and cover are high. It has been previously observed in neighboring areas that the maximum biomass of organisms associated (flora and fauna) with this macroalgae occurs when the average length of the thallus (*N* = 240) is approximately 12.0 cm in September (*N* = 20) rather than when it reaches its maximum length (16.3 cm) in October (*N* = 20) [57]. This suggests that the assemblage of hydrozoan epibionts of *A. spicifera* is changing in response to increased temperatures in the warm season. However, although the water temperature is a significant factor influencing the composition and structure of hydrozoan assemblages at a given location, the potential influence of other abiotic factors and biotic interactions cannot be discounted [48,56,67,68].

## 5. Conclusions

At UABCS’ Pichilingue Pier, the similarity in epibiont hydrozoan community structure (cover and compositions) is defined by the dominant taxa, and the differences in both seasonality and thallus sections are due to the high coverage of rare taxa. The abiotic and biotic conditions in the warm season favor the growth of hydrozoan taxa, in contrast to *A. spicifera*, which exhibits a reduction in the average size of its thallus. Consequently, most hydrozoan colonization occurs in a single taxon per thallus and with an affinity for the basal area, therefore reducing competition between species.

According to this and previous studies on non-native algae in La Paz Bay (cf. [13,14]), it is recommended that a sampling design monitoring of this macroalga and its epibionts be conducted throughout the warm, cold, and transitional seasons for the periodic changes in surface temperature and the entrance and retirement of tropical waters in the region during these seasons [21,23]. As demonstrated by the sampling effort and completeness analysis, the minimum number of thalli to be analyzed per season should be 30 to ensure comprehensive data collection. Each thallus should be analyzed in its entirety, including all localities where the macroalga have been recorded. This is because macroalgae of the same species can differ between localities (e.g., in size and shape), depending on the conditions under which they are exposed [64]. In addition, sampling for morphological and genetic analyses should be considered for accurate knowledge of the epibiont species and timely detection of non-native species. These macroalgae have no epibionts that threaten the region’s biological diversity.

## Figures and Tables

**Table 1:** Systematic list and means percentage cover of the hydrozoan (Hydrozoa) epibionts of *Acanthophora spicifera*. Presence of hydrozoans on each section of thalli: B = basal section, M = middle section, and A = apical section. Values without standard deviation correspond to a single sample.

**Taxa**	**Warm Season (35 Ups, 28 °C)**	**Cold Season (35 Ups, 25 °C)**	**Section**
Class Hydrozoa Owen, 1843			
Subclass Hydroidolina Collins, 2000			
Superorder “Anthoathecata” Cornelius, 1992			
*Bimeria vestita* Wright, 1859	1.5		B
*Bougainvillia muscus* (Allman, 1863)	9.9 ± 5.3	5.0	M, B
Capitata (Indet.)	14.3		B
*Corydendrium* sp. (Indet.)		3.4	B
*Turritopsis* sp. (Indet.)	13.7		B
Superorder Leptothecata Cornelius, 1992			
*Clytia* cf*. gracilis* (M. Sars, 1851)	1.6		A
*Clytia linearis* (Thornely, 1900)	5.7 ± 3.1	2.9 ± 1.2	A, M, B
Lafoeidae (Indet.)	2.8		M
*Obelia* cf*. dichotoma* (Linnaeus, 1758)	11.8 ± 12.8	4.6 ± 3.8	A, M, B
*Obelia oxydentata* Stechow, 1914	3.9		A, B
*Ventromma halecioides* (Alder, 1859)	8.1		M, B

## Data Availability

The authors will share the database supporting this research as Supplementary Material.

## Supplementary Materials

The following supporting information can be downloaded at: https://www.mdpi.com/article/10.3390/biology14010044/s1, Table S1: Hydrozoan epibionts of *Acanthophora spicifera* at UABCS Pichilingue Pier, La Paz Bay, Baja California Sur, Mexico.
